# Positional plagiocephaly following ventriculoperitoneal shunting in neonates and infancy—how serious is it?

**DOI:** 10.1007/s00381-016-3275-z

**Published:** 2016-11-15

**Authors:** Stuart A. G. Roberts, Joseph D. Symonds, Reema Chawla, Emma Toman, Jonathan Bishop, Guirish A. Solanki

**Affiliations:** 1 Department of Paediatric Neurosurgery, Birmingham Children’s Hospital, Birmingham, West Midlands UK; 2 The Computational, Cognitive and Clinical Neuroimaging Laboratory (C3NL), The Hammersmith Hospital, 3rd Floor, Burlington Danes Building, Du Cane Road, W12 0NN London, UK; 3 Fraser of Allander Neurosciences Unit, Royal Hospital for Children, G51 4TF Glasgow, UK; 4 National Institute for Health Research (NIHR), Surgical Reconstruction & Microbiology Research Centre (SRMRC), Birmingham, UK; 5 Birmingham Clinical Trials Unit, Robert Aitken Institute, University of Birmingham, B15 2TT Edgbaston, UK

**Keywords:** Ventriculoperitoneal, Plagiocephaly, Ventriculostomy

## Abstract

**Purpose:**

We test the hypothesis that ventriculoperitoneal (VP) shunt insertion significantly increases contralateral positional plagiocephaly.

**Methods:**

We reviewed 339 children who had a VP shunt inserted at Birmingham Children’s Hospital between 2006 and 2013, noting laterality of shunt insertion and frontal or occipital position. We ascertained the presence of post-operative positional plagiocephaly using the cranial vault asymmetry index. Multinomial logistic regression modelling was used to examine relationships between plagiocephaly, shunt position, gender and age. Adjusted odds and risk ratios for effect of variables on plagiocephaly were calculated.

**Results:**

Children with occipital VP shunts are at significant risk of developing contralateral positional plagiocephaly, particularly in the first 12 months of life.

**Conclusions:**

We recommend careful follow-up and advice regarding head positioning following surgery. There should be consideration for active monitoring to avoid plagiocephaly, including physiotherapy and health visitor interventions. Endoscopic third ventriculostomy in selected cases or anterior shunt placement could be considered. A larger national study would be of interest to evaluate the extent of an otherwise correctable problem.

## Introduction

**Fig. 1:**
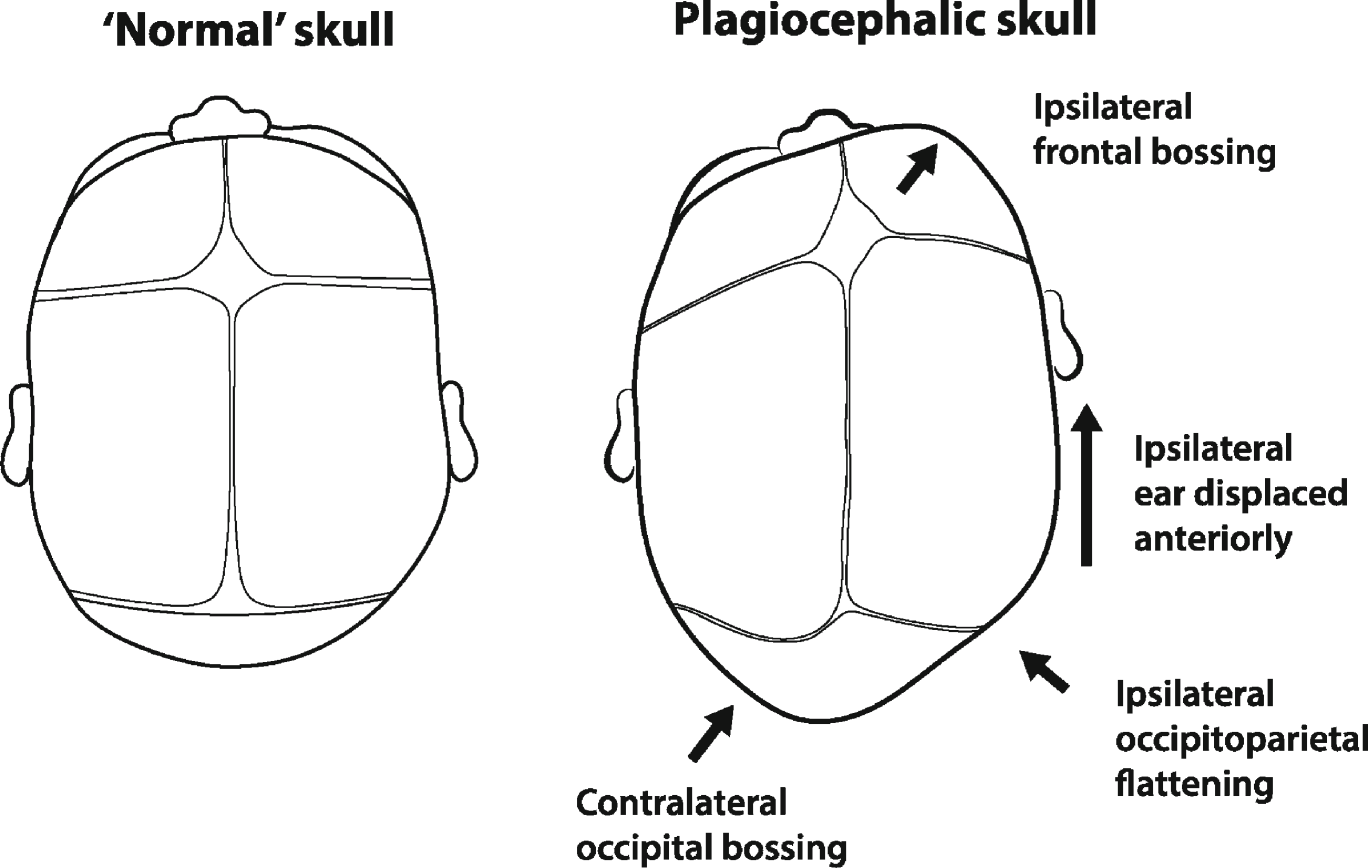
Features of positional plagiocephaly compared with a normal skull. The side with plagiocephaly is characterised by occipitoparietal flattening and contralateral occipital bossing, with anterior displacement of the ear and frontal bossing later signs

Plagiocephaly (ICD 10 Q67.3) means *oblique head* (Greek *plagios* = oblique; *kephale* = head) [1]. The term encompasses cranial asymmetry arising from synostotic and non-synostotic causes. Synostotic plagiocephaly results from premature closure of the skull sutures (anterior due to unicoronal synostosis; posterior due to lambdoid synostosis). Positional or deformational plagiocephaly is non-synostotic, resulting from prolonged recumbence of the child creating pressure on the skull. Some babies have a tendency to lie on one side more and this side flattens and shifts forward creating a parallelogram shape when viewed from the vertex. This typically results in unilateral flattening of the parieto-occipital region, anterior advancement of the ipsilateral ear, and anterior displacement of the ipsilateral forehead (Fig. 1). Argenta proposed a clinical classification of positional plagiocepahly [2]. In type 1, the asymmetry is completely limited to the back of the skull, while types 2–5 involve progressive degrees of facial and cranial asymmetry.

Postnatal positioning contributes to development of positional plagiocephaly, with prevalence increasing since the 1990s [3]. It is generally accepted that this increase in prevalence has been a result of public health campaigns that have encouraged parents to place infants supine to sleep [4]. Case-control studies have demonstrated prone sleep position to be a risk factor for sudden unexpected death in infancy (SUDI) [5]. The incidence of SUDI has fallen significantly since the introduction of these campaigns [6]. A recent cross-sectional study of 440 healthy Canadian children found the incidence of positional plagiocephaly to be 46.6 %, 77.3 % of who had type 1 or type 2 plagiocephaly, and 78.3 % were clinically described as mild cases [7].

Positional plagiocephaly is generally believed to be benign. However, it may be associated with neurodevelopmental impairments. Miller interviewed 63 families in whom a child was affected by positional plagiocephaly and found that 39.7 % of children with positional plagiocephaly received additional education support, compared with 7.7 % of sibling-controls (*X* ^2^ = 21.24) [8].

Current treatments for positional plagiocephaly include a ‘wait and see’ approach, parental education regarding head positioning, cranial orthoses, surgery or a combination of these [9–11].

Hydrocephalus results from an imbalance between cerebrospinal fluid production and absorption into the bloodstream. It is one of the most common pediatric neurosurgical conditions [12]. Longitudinal studies show ventriculoperitoneal (VP) shunting has resulted in improved outcomes although neurological function is variable in affected children [13]. VP shunts are associated with several complications, including infection, shunt migration, and hemorrhage [14]. Positional plagiocephaly as a result of VP shunt insertion has not previously been described or quantified.

We hypothesize contralateral plagiocephaly secondary to VP shunt insertion will be increased in the neonate and infancy. This study aims to assess development of post-operative contralateral posterior plagiocephaly in a pediatric setting following insertion of a VP shunt. We aim to characterise this entity, evaluating frequency and severity.

## Methods

We undertook a retrospective cohort study within the Department of Paediatric Neurosurgery, Birmingham Children’s Hospital between 2006 and 2013. We included children aged 0–16 years from our departmental surgical database with at least one follow-up scan. We excluded children with incomplete data, incomplete post-operative imaging; bilateral ventriculoperitoneal shunts and pre-existing plagiocephaly (see below). The surgical database contained 455 children. One hundred sixteen children were excluded due to a lack of imaging. Three hundred thirty-nine children had a VP shunt and satisfactory post-operative imaging. All children had a pre-operative baseline CT scan.

**Fig. 2:**
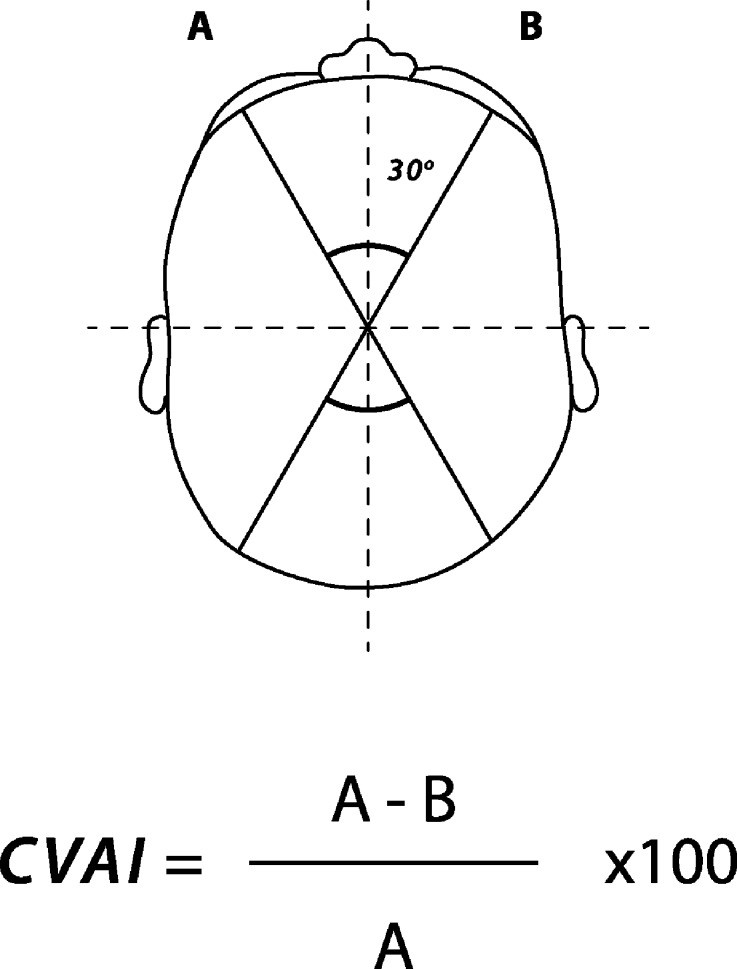
The cranial vault asymmetry index (CVAI) is the difference between the lengths of two diagonals measured 30 degrees from midline, divided by the larger of the two diagonals. Multiplication by 100 results in a percentage. Using an index instead of a single measurement normalizes measurements allowing head shapes of various sizes to be compared, proportionally. A symmetrical head would have a CVAI of 0 %, while a head is considered asymmetric if the CVAI is ±3.5 %

For analysis we stratified age groups as 0–1 month (neonate), 1–12 months (infant), 1–3 years, 3–5 years, 5–12 years and 12–16 years. We recorded demographics, date of operation, laterality, position of shunt (frontal/occipital), and presence/site of plagiocephaly. We used the cranial vault asymmetry index (CVAI) to assess plagiocephaly in pre-operative and post-operative images (Fig. 2)—permitting exclusion of pre-existing plagiocephaly. This is the difference between the lengths of two diagonals measured 30 degrees from midline, divided by the larger of the two diagonals, with this value multiplied by 100, creating a percentage. Using an index instead of a single measurement normalises measurements allowing head shapes of various sizes to be compared, proportionally. A symmetrical head would have a CVAI of 0 %, while a head is considered asymmetric if the CVAI is ±3.5 %. Although somewhat arbitrary, it is widely used while other assessments of plagiocephaly are based on clinical experience, parental concerns and clinical perception [15]. We performed a single post-operative CVAI measurement. This was taken from the first scan prior to follow-up although timing of this was not consistent across the period.

## Analysis

We analyzed the raw data for significance using SPSS (IBM, v22.0). We performed Pearson’s chi-squared test (with Yates’ continuity correction) of independence and the Cochran-Armitage test for trend. Additionally, we performed odds and risk ratios for the dataset.

Multinomial logistic regression modelling was used in order to examine the relationships between plagiocephaly, shunt position, gender and age. Adjusted odds and risk ratios for effect of variables on plagiocephaly were then calculated. In the model, we specified ‘Posterior’ as the reference level for the occipital shunt position, ‘Neonate’ as the reference level for age, and ‘Female’ as the reference level for gender. Confidence intervals were obtained using the percentile method on the output from 9999 bootstrap resamples. Goodness of fit statistics indicates an adequate fit of our model to the data, with an unweighted sum of square error of 55.67 and associated *p* value of 0.64.

## Results

We show that 54 % of shunt-associated plagiocephaly occurs in the first year. Eighty-one children (23.9 %) developed positional plagiocephaly following VP shunt insertion. Of those, 54 children (54 %) were boys and 27 (33 %) were girls (*p* 0.110).

Assessing development of plagiocepahly by age at time of identification showed 17 % occurred in neonates and as much as 37 % in infancy. Seven percent occurred in 1–3-year group, 10 % in 3–5-year group, 25 % in the 5–12-year group and 4 % in 12–16-year group.

Positional plagiocephaly is significantly more common following occipital shunt placement. Four children (5 %) developed plagiocephaly following frontal shunt placement, compared to 77 (95 %) following occipital shunt placement, (*p* 0.046) (Table 1). The risk ratio of plagiocephaly is 0.39 for children receiving frontal shunt compared to an occipital shunt. The corresponding odds ratio is 0.32.

**Table 1:** Data summarized by presence or absence of positional plagiocephaly. This is significantly more common following occipital shunt placement. The *p* values correspond to Pearson’s chi-squared test (with Yates’ continuity correction) of independence

	Plagiocephaly		*p* value
	Absent	Present	
Shunt position			
Frontal	36 (14 %)	4 (5 %)	0.046
Occipital	222 (86 %)	77 (95 %)	
Gender			
Female	114 (44 %)	27 (33 %)	0.110
Male	144 (56 %)	54 (67 %)	
Age			
Neonate	25 (10 %)	14 (17 %)	4*e*–05^a^
Infant	49 (19 %)	30 (37 %)	
1–3 years	33 (13 %)	6 (7 %)	
3–5 years	23 (9 %)	8 (10 %)	
5–12 years	83 (32 %)	20 (25 %)	
12–16 years	45 (19 %)	3 (4 %)	

^a^Via Cochran-Armitage test for trend

We examined associations between plagiocephaly with shunt position, gender and age using a logistic regression model. This permits us to examine adjusted odds and risk ratios for the effect of these variables on plagiocephaly. We tabulate the estimates and standard errors from the model output (Table 2).

**Table 2:** Output from logistic regression modelling of data examining associations between positional plagiocephaly with shunt position, gender and age. This permits us to examine adjusted odds and risk ratios for the effect of these variables on presence of plagiocephaly

			*Dependent variable*	
			*Plagiocephaly*	
Variable	Level	Estimate	Std. error	*p* value
Age	Infant	0.122	0.415	0.768
	1–3 years	−1.105	0.569	0.052
	3–5 years	−0.531	0.539	0.324
	5–12 years	−0.804	0.424	0.580
	12–16 years	−2.258	0.691	0.001
Gender	Male	0.720	0.283	0.011
Shunt	Frontal	−1.203	0.562	0.032
Constant		−0.919	0.280	0.016
Observations	339			
*R* ^2^	0.149			
Χ^2^	35.418 (df = 7)	Pr (>Χ^2^) < 0.0001		

When we adjust for age and gender, we obtain an adjusted odds ratio of 0.300 with 95 % confidence interval (0.085, 0.816) for the presence of plagiocephaly in children receiving frontal shunt compared to an occipital shunt. Assuming all other variables are held fixed, then children receiving frontal shunt have a 70 % reduction in the odds of post-operative plagiocephaly. We also obtained risk ratios and corresponding statistics [16] (Table 3).

**Table 3:** Table of adjusted odds and risk ratios for developing positional plagiocephaly. Confidence intervals are obtained using the percentile method on the output from 9999 bootstrap resamples. Children receiving a frontal shunt have a 70 % reduction in the odds of post-operative plagiocephaly. Approximately five children need to receive a frontal shunt for one to benefit. Children with frontal shunts experience, on average, a 45 % reduction in risk ratio of becoming plagiocephalic compared to those receiving occipital shunts

Statistic	Estimate	95 % confidence interval
Odds ratio	0.300	(0.085, 0.816)
Risk difference	0.202	(0.195, 0.210)
Risk ratio	0.549	(0.534, 0.563)
NNT	4.947	(4.773, 5.131)
Risk ratio reduction	45.1 %	(43.7, 46.6 %)

There is strong evidence that children receiving frontal shunts have considerably lower incidence of plagiocephaly than those receiving an occipital shunt. Approximately five children need to receive a frontal shunt for one to benefit (i.e. not become plagiocephalic) compared with children receiving an occipital shunt (Table 3). Children receiving frontal shunts experience, on average, a 45 % reduction in risk ratio of becoming plagiocephalic compared to those receiving an occipital shunt.

## Age groups

Older children are at lower risk of post-operative plagiocephaly. Examining model output for age group, we find there is no statistically significant difference between neonates and infants, or neonates and those aged 3–5 years. We do, however, find that there are statistically significant differences in probabilities of becoming plagiocephalic between neonates and 12–16 year olds. Differences between neonates and 1–3 year olds and between neonates and 5–12 year olds are of borderline significance.

Again, using logistic regression to adjust for the effect of shunt position and gender, we obtain an adjusted odds ratio of 0.105 (with 95 % confidence interval (0.022 to 0.363)) for the presence of plagiocephaly in children aged 12–16 years compared to neonates. Assuming other variables are fixed, then children aged 12–16 years have an 89 % reduction in the odds of becoming plagiocephalic following a VP shunt, compared to neonates.

## Gender

Boys are more likely to develop shunt-associated plagiocephaly than girls. Adjusting for the effect of shunt position and age, we obtain an adjusted odds ratio of 2.054 (with 95 % confidence interval (1.189, 3.618)) for the presence of plagiocephaly in boys compared to girls. Assuming all other variables are held fixed, then boys have a 105 % increase in the odds of becoming plagiocephalic.

## Discussion

We show that positional plagiocephaly is associated with VP shunt insertion. This association is significantly increased in VP shunt placement in the first 12 months. Though positional plagiocephaly has been shown to be common in healthy children, we are confident that VP shunting plays a role in these patients since we have found plagiocephaly consistently occurs contralateral to the shunt position, whereas idiopathic plagiocephaly typically has a right-sided bias. The most likely mechanism for causation is that infants with VP shunts are positioned on the contralateral side post-operatively and following discharge.

We acknowledge some limitations with our results. Although we have 339 children and 81 instances of positional plagiocephaly, when stratified by shunt position, we only have four children both plagiocephalic and with a frontal shunt. Hence, we attempted to estimate a model parameter with only four pieces of data.

We used the CVAI with electronic measurement for pre- and post-operative CT scans, which allowed highly accurate measurements. CT is not commonly used to assess non-synostotic plagiocephaly since there is a hazard associated with application of ionizing radiation in the growing skull [17]. High-resolution ultrasound has been shown to be a useful imaging adjunct in those requiring imaging [18]. While accurate and without ionizing radiation, MRI in pediatric populations often requires sedation or anesthesia [19]. As children had pre-existing CT, a further MRI was not indicated. More commonly, a diagnosis of positional plagiocephaly is made clinically, or with external measuring devices [15]. As this was a study in children with post-operative imaging, we believe it permitted an accurate assessment of CVAI. This study only examines contralateral plagiocephaly. Ipsilateral or bilateral instances would require further study. Our study is single-centre study, opening it to bias regarding operator technique and local practices. A multi-centre approach would mitigate this in future work.

In the light of our findings, families should be counselled to anticipate plagiocephaly following surgery. Parents could be advised regarding positioning the head following surgery. This is also relevant in nursing particularly infants both in hospital and on discharge, where it is shown that positioning is still largely influenced by guidelines to prevent SUDI [20]. This creates a challenge since those children most at risk of positional plagiocephaly can less ably be repositioned. The back to sleep campaign was enormously successful and there is no suggestion this advice should not be followed; however, turning the head to the opposite side or alternating head position may reduce development of plagiocephaly post VP shunting.

Our results suggest increased insertion of frontal shunts may reduce plagiocephaly rates; however, anecdotal experience from our centre suggests frontal shunting in neonates and infants may cause greater long-term disconnection and ventricular catheter extrusion. As the head grows against a fixed valve, the catheter is pulled between the retro-auricular valve and the ventricular portion. Additionally, overgrowth of the bone over the reservoir has been observed, making it impossible to test for ventricular catheter patency or permit emergency aspiration of cerebrospinal fluid. Cosmetics of frontal shunts may also be an issue in some.

A long-term analysis of pediatric VP shunt placement has shown a relatively high rate of complications. Requirement for shunt revision was as late as 17 years after initial placement with 84.5 % of the patients requiring one or more shunt revision [21].

There is some evidence that positional plagiocephaly may be associated with neurodevelopmental impairments. Studies have shown that children were more likely to require additional support such as speech therapy, occupational therapy and physiotherapy [8]. Other studies have used parental questionnaires and found that parents of children with plagiocephaly perceived developmental delays, particularly head lag and delays in rolling over [22]. Even if these associations are real, the direction of causation is not clear [23].

In the light of the above uncertainties, the most generally accepted approach to positional plagiocephaly following VP shunting remains conservative management [10, 20]. Such an approach would begin with pre-operative parental education. Additional support may be required for families from lower socioeconomic backgrounds [24]. Post-operatively, appropriate alternating of head position should be practised while respecting existing SUDI mitigation guidelines, with appropriate advice given to parents on discharge. There is evidence in persistent cases that helmet orthoses are extremely effective. However, these have an associated cost and may be associated with stigma [11].

## Conclusions and recommendations

We have shown that children with occipital VP shunts are at increased risk of contralateral positional plagiocephaly, particularly in those operated in the first 12 months of age. This is likely benign and unlikely to be associated with neurodevelopmental complications. A longitudinal study is planned to evaluate if infant head shape normalizes with growth. We believe simple advice early on could allay later concerns such as parental anxiety or leaving older children open to bullying as a result of their head shape. In all children with occipital VP shunt placement, we recommend careful follow-up and strong advice regarding head positioning (for example alternating from right to left on consecutive nights) following surgery. There should be consideration for active monitoring to avoid plagiocephaly, including physiotherapy and health visitor interventions. Endoscopic third ventriculostomy in selected cases or frontal shunt placement could be considered. A larger national study would be of interest to evaluate the extent of an otherwise correctable problem.
